# Bacterial Communities in Alkaline Saline Soils Amended with Young Maize Plants or Its (Hemi)Cellulose Fraction

**DOI:** 10.3390/microorganisms9061297

**Published:** 2021-06-15

**Authors:** Valentín Pérez-Hernández, Mario Hernández-Guzmán, Marco Luna-Guido, Yendi E. Navarro-Noya, Elda M. Romero-Tepal, Luc Dendooven

**Affiliations:** 1 Laboratory of Soil Ecology, Department of Chemistry and Biochemistry, Instituto Tecnológico de Tuxtla-Gutiérrez, Tecnológico Nacional de México, Tuxtla Gutiérrez, Chiapas 29050, Mexico; vperezhdez@hotmail.com; 2 Laboratory of Soil Ecology, Cinvestav, Mexico City 07360, Mexico; hg.maryo@gmail.com (M.H.-G.); mluna@cinvestav.mx (M.L.-G.); elda.romero.tepal@gmail.com (E.M.R.-T.); 3 Centro de Investigación en Ciencias Biológicas, Universidad Autónoma de Tlaxcala, Tlaxcala 90070, Mexico; nyendi@hotmail.com

**Keywords:** pyrosequencing, lignocellulose degradation, PICRUSt prediction, Texcoco soil

## Abstract

We studied three soils of the former lake Texcoco with different electrolytic conductivity (1.9 dS m^−1^, 17.3 dS m^−1^, and 33.4 dS m^−1^) and pH (9.3, 10.4, and 10.3) amended with young maize plants and their neutral detergent fibre (NDF) fraction and aerobically incubated in the laboratory for 14 days while the soil bacterial community structure was monitored by means of 454-pyrosequencing of their 16S rRNA marker gene. We identified specific bacterial groups that showed adaptability to soil salinity, i.e., *Prauseria* in soil amended with young maize plants and *Marinobacter* in soil amended with NDF. An increase in soil salinity (17.3 dS m^−1^, 33.4 dS m^−1^) showed more bacterial genera enriched than soil with low salinity (1.9 dS m^−1^). Functional prediction showed that members of Alfa-, Gamma-, and Deltaproteobacteria, which are known to adapt to extreme conditions, such as salinity and low nutrient soil content, were involved in the lignocellulose degradation, e.g., *Marinimicrobium* and *Pseudomonas* as cellulose degraders, and *Halomonas* and *Methylobacterium* as lignin degraders. This research showed that the taxonomic annotation and their functional prediction both highlighted keystone bacterial groups with the ability to degrade complex C-compounds, such as lignin and (hemi)cellulose, in the extreme saline-alkaline soil of the former Lake of Texcoco.

## 1. Introduction

High salinity is one of the major limitations for using soil for agriculture practices, and it is estimated that, in the world, approximately 351.2 million ha are saline and 581 million ha sodic [1]. Salinity has a profound effect on the structure [2], nutrient content [3], and water movement in soils [2], but it also affects the microbial [4] and plant diversity [5]. In Mexico and central America, approximately two million ha are saline. They are natural saline (e.g., Solonchak) or as the result of anthropogenic activity (e.g., soil irrigation) [1]. Lake Texcoco, which originally surrounded the Aztec city of Tenochtitlan (currently Mexico City), was drained mostly leaving a soil with little or no vegetation exposed to wind erosion. Soil in some parts of the former lake Texcoco are extremely alkaline saline with pH reaching 10.5 and an electrolytic conductivity (EC) of 200 dS m^−1^ [6]. The high soil pH and EC are the result of brackish groundwater that reaches the soil surface and then evaporates. The former lakebed has been drained for different periods, so, in certain areas, the EC (<2 dS m^−1^) and pH (<10) has dropped, i.e., but, in other parts, the pH remains >10 and the EC > 100 dS m^−1^. The variation in salinity and alkalinity allows to study how changes in EC and pH affects different soil processes, such as mineralization of organic material [7] or denitrification [8], and the microbial community, i.e., Bacteria and Archaea [9].

The degradation of organic material occurs in the Texcoco soil, although high salinity is known to inhibit mineralization and cellulose degradation. A study with ^14^C labelled young maize plants showed that the plants and neutral detergent fibre (NDF) fraction (mostly (hemi)cellulose) were mineralized in a soil with EC 32.7 dS m^−1^ and pH 9.8 [7]. However, it remained unclear as to which organisms were involved in the degradation of the organic material in the extreme alkaline saline soil. Under these conditions, isolation techniques proved the presence of specialized bacterial cellulose degraders, e.g., *Cellulomonas bogoriensis* and *Nocardiopsis dassonvillei* [10]. Nevertheless, the limitations in isolation techniques used, i.e., only a small number of microorganisms can be cultured, gives only a glimpse of the diversity and functionality of the microorganisms in the soil environment. In a previous study, one soil of the former lake Texcoco was flooded and the reduction in soil EC did not affect the bacterial capacity to mineralize organic material applied, but it did affect specific bacterial groups [11].

The aim of this study was to determine the effect of salinity and alkalinity on the bacterial community structure and their functionality when young maize plants or their NDF fraction was applied to three different soils from Texcoco with varying alkalinity and salinity. Soil was taken from the former Texcoco lakebed and then amended with young maize plants or their NDF fraction, mostly (hemi)cellulose, and aerobically incubated for 14 days while changes in the bacterial community structure were monitored by means of 454 pyrosequencing of the 16S rRNA marker gene.

## 2. Materials and Methods

### 2.1. Cultivation of the Maize and Neutral Detergent Extraction

Details of how the maize (*Zea mays* L.) was cultivated and its characteristics can be found in the study conducted by Ramírez-Villanueva et al. [12]. Briefly, maize seeds were surface sterilized and germinated on 0.8% agar. Maize seedlings that had formed 2 cm roots were placed on sterilized and C-free vermiculite in the growth chamber and watered regularly with a nutritive Steiner solution [13]. Twenty-five days after planting, the maize plants were harvested, air-dried, and characterised.

The maize plants were characterised and fractionated [14,15] to obtain the neutral detergent fibre (NDF) fraction, as described in Ramírez-Villanueva et al. [12] (Table S1). The NDF fraction is obtained after hot extraction with a neutral detergent solution that removes the “soluble” part of the maize plants, leaving most of the cell wall constituents, i.e., (hemi)cellulose plus some lignin. The young maize plants contained 599 ± 6.5 g kg^−1^ soluble fraction, 311 ± 8.0 g kg^−1^ (hemi)cellulose, 25 ± 1.1 g kg^−1^ lignin, and the rest ash, while the NDF fraction contained 776 ± 15.4 g kg^−1^ (hemi)cellulose, 63 ± 2.8 g kg^−1^ lignin, and 155 ± 2.9 g kg^−1^ ash.

### 2.2. Soil Sampling

The soil was sampled from the lakebed of the former lake Texcoco at three locations (Figure S1). At the three locations, three 400-m^2^ areas were defined. In each area, the soil was sampled 20 times by augering the 0–20 cm layer. The 20 soil samples of each area were pooled separately. As such, nine soil samples were obtained, i.e., from three locations (*n* = 3) in triplicate (*n* = 3). The soil samples were taken on ice to the laboratory, 5-mm sieved separately, and then characterised (Table 1). This field-based replication was maintained in the laboratory experiment to avoid pseudoreplication [16].

The particle size distribution was determined by the hydrometer method [17], total organic carbon was measured with a carbon analyzer TOC-VCSN (Shimadzu, Canby, OR, USA), inorganic C, as described by Bundy and Bremner [18], and total nitrogen by the method outlined by Kjeldahl [19]. The water holding capacity was determined by the gravimetric method, EC, as reported by Rhoades et al. [20], and the pH was measured in a suspension sediment-H_2_O using a glass electrode [21].

### 2.3. Experimental Design and Microcosms Setup

The aerobic incubation was done, as developed at the soil science department in Rothamsted, e.g., Inubushi et al. [22]. The soil was incubated for 14 days, as previous research with soil of Texcoco [7] had found that the mineralization of ^14^C-labelled maize residue and its NDF fraction was most accentuated in the first days of an aerobic incubation.

The soil was separately pre-incubated in drums containing 1-L jar with 1 M NaOH to trap evolved CO_2_ and 1-L jar with distilled water to avoid soil desiccation for a week. After seven days, 15 sub-samples of 25 g from each soil (*n* = 9) were added separately to 120-mL glass flasks. Five samples were amended with young maize plants, five with the NDF fraction of the young maize plants and five were left unamended. The amount of organic material applied was such that 2 g C kg^−1^ was applied to soil. The soil of each treatment was mixed, and the glass flasks were placed separately in 1-L glass jars with 10 mL distilled water to avoid desiccation and containing a 25-mL flask with 20 mL 1 M NaOH to trap the evolved CO_2_. The glass jars were closed airtight and stored in the dark at 22 ± 2 °C (the mean annual temperature of the studied sites) for 14 days. Additionally, 15 flasks without soil, i.e., only containing NaOH solution and water, were incubated also for 14 days. These flasks were used to account for the CO_2_ trapped from the environment. After one, three, seven, and 14 days, a jar from each soil (*n* = 3), replicate (*n* = 3), and treatment (*n* = 3) were selected at random, opened and the flasks with 1M NaOH removed, and the soil was stored at −20 °C pending DNA extraction. The 25-mL flasks with 1M NaOH were removed from the flasks, closed airtight, and stored until analysed for the CO_2_ trapped in the 1M NaOH. Additionally, three flasks without soil, but with a 25-mL flask with NaOH and water were opened, and the 25-mL flasks removed, closed airtight and stored. At day 7, all of the remaining flasks with soil or without soil were opened and then aired to avoid anaerobic conditions. After 10 min., all flasks were closed air-tight and then incubated for another seven days.

### 2.4. DNA Extraction and PCR Amplification of Bacterial 16S rRNA Genes

Metagenomic DNA was extracted with three different techniques twice from 0.5 g soil and pooled. As such, a total of 3 g soil was extracted for DNA per plot (*n* = 3). Each soil sample was washed with 0.15 M sodium pyrophosphate and 0.15 M phosphate buffer pH 8 to remove the fulvic and humic acids [23]. Three different techniques were then used to extract the DNA from the washed soil. The first method was developed by Valenzuela-Encinas et al. [24] and it consisted in a chemical and thermal shock for cell lysis. The second method, in which cells were lysed enzymatically, was developed by Sambrook and Rusell [25], while the third method used a detergent solution for cell lysis (chemical method), as described by Hoffman and Winston [26].

The V1-V6 region of the 16S rRNA bacterial genes was amplified with 10-pb barcoded primers 8-F (5′-AGA GTT TGA TCI TGG CTC A-3′) and 949-R (5′-CCG TCW ATT KCT TTG AGT T-3′) and containing the A and B 454 FLX adapters [27]. The PCR reactions were done, as previously described by Navarro-Noya et al. [27]. The product of five reactions of each metagenomic DNA sample was pooled to minimise the PCR bias and constituted a single library [28]. All of the pyrosequencing libraries were purified using the DNA Clean & Concentrator purification kit, as recommended by the manufacturer (Zymo Research, Irvine, CA, USA), and quantified using the PicoGreen^®^ dsDNA assay (Invitrogen, Carlsbad, USA) and the NanoDrop^TM^ 3300 Fluorospectrometer (Thermo Fisher Scientific Inc., Suwanee, CA, USA). Sequencing was done by Macrogen Inc. (DNA Sequencing Service, Seoul, Korea) using a Roche 454 GS-FLX Plus System (Roche, Mannheim, Germany).

### 2.5. Analysis of Pyrosequencing Data

The QIIME version 1.9.0 software was used to analyse the pyrosequencing data [29]. The low-quality reads were eliminated from the data sets, i.e., quality score < 25, containing homopolymers > 6, length < 400 nt, and containing errors in primers and barcodes. Operational taxonomic units (OTUs) were determined at 97% similarity level with the UCLUST algorithm [30]. Chimeras were identified and removed from the data sets using Chimera Slayer [31]. The sequence alignments were done against the GreenGenes core set and using representative sequences of each OTU using PyNAST and filtered at a threshold of 75% [32]. Rarefaction was done using the alpha_rarefaction.py command within QIIME v1.9.0 using default settings.

The functional potential of bacterial communities in soil samples were predicted using the phylogenetic investigation of communities by reconstruction of unobserved states (PICRUSt v1.1.2) pipeline [33]. The functional profile was conducted using the “Kyoto Encyclopedia of genes and genomes” (KEGG) and genes that were related to the degradation of cellulose, lignin, hemicellulose, and cello-oligosaccharides were analysed [34] (Table S2) using *metagenome_contribution.py* command. In addition, the functional annotation of prokaryotic taxa v1.2.4 (FAPROTAX) [35] was used to investigate functional pathways in the environment, e.g., C and N cycles.

The bacterial diversity was determined using the equivalent Hill numbers at different *q* orders (at *q* = 0, 1, and 2) and they were calculated using the raw count dataset at the genus taxonomic level, as described in Ma and Li [36].

### 2.6. Statistical Analysis

Statistical analyses were done within the R software v4.0.2. [37]. A one-way ANOVA with Tukey post hoc analysis was used to determine significant differences in soil physicochemical characteristics (*p* < 0.05). Heatmaps of the relative abundance of the bacterial groups were constructed with the pheatmap package [38]. The ALDEx2 package v1.21.1. was used to transform the sequence counts data using the centered log-ratio (clr) transform test returned by the aldex.clr argument [39] prior to ordination analysis. Principal component analysis (PCA) was used to explore the effect of treatment on the bacterial groups using FactoMineR package v2.3 [40]. The permutational multivariate analyses of variance (perMANOVA) test was used to determine the effect of treatments (unamended soil or soil amended with young maize plants or its NDF fraction), soil electrolytic conductivity value (1.9 dS m^−1^, 17.3 dS m^−1^ and 33.4 dS m^−1^), and incubation time (one, three, seven, 14 days) using Vegan v2.5 [41]. The non-parametric Kruskal–Wallis test with Benjamini–Hochberg corrected *p* value (aldex.kw argument within ALDEx2 package) was used to determine the effect of electrolytic conductivity (EC) value and soil treatment on the bacterial groups using clr-transformed data. The differential abundance analysis between treatments (soil amended with young maize plants or its NDF) when compared to the unamended control soil was done with DESeq2 v1.28.1 package [42]. Only bacterial groups with assigned reads counts >10 were considered for differential abundance analysis with DESeq2.

## 3. Results

### 3.1. Carbon Mineralization

The application of young maize plants increased the emitted CO_2_ in each soil, but not when the NDF fraction was added (Figure S2). After 14 days, the CO_2_ that was emitted in the young maize plants-amended soil was significantly higher in soil with EC 1.9 dS m^−1^ than in the other two soils (*p* < 0.05).

### 3.2. Sequencing Results and Bacterial Diversity

Overall, 369,016 good quality sequences were retrieved from the soil representing 8478 OTUs. The number of sequences retrieved was sufficient, as the rarefication curves were asymptotic (Figure S3). As such, an increase in the number of sequences analysed would only yield a limited number of OTUs more. The soil of the former lakebed was characterised by a large variation in the number of bacterial groups at different taxonomical levels. Overall, 31 different phyla, 93 classes, 142 orders, and 194 genera were detected in the soil.

At the onset of the experiment and after 14 days, the effective numbers of bacterial genera *q* = 0 (species richness, ^0^D) and *q* = 1 (typical genera, ^1^D) in unamended soil decreased with increased EC (Figure S4). The application of young maize plants or the NDF fraction decreased the ^0^D and ^1^D value with the lowest values being found in the NDF fraction amended soil. The soil with the highest EC had the highest ^2^D in the unamended soil and ^2^D decreased in all treatments over time.

### 3.3. Bacterial Community Structure in the Unamended Soil

**Figure 1:**
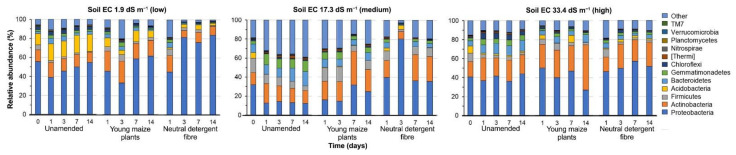

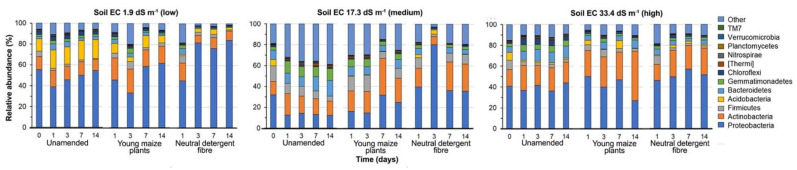
Changes in the relative abundance (%) of bacterial phyla in the three soils from the former lake Texcoco with different electrolytic conductivity (EC) left unamended or amended with young maize plants or their neutral detergent fiber (NDF) fraction at the onset of the experiment (0) and incubated aerobically at 22 ± 2 °C for one (1), three (3), seven (7), or 14 days (14).

**Figure 2:**
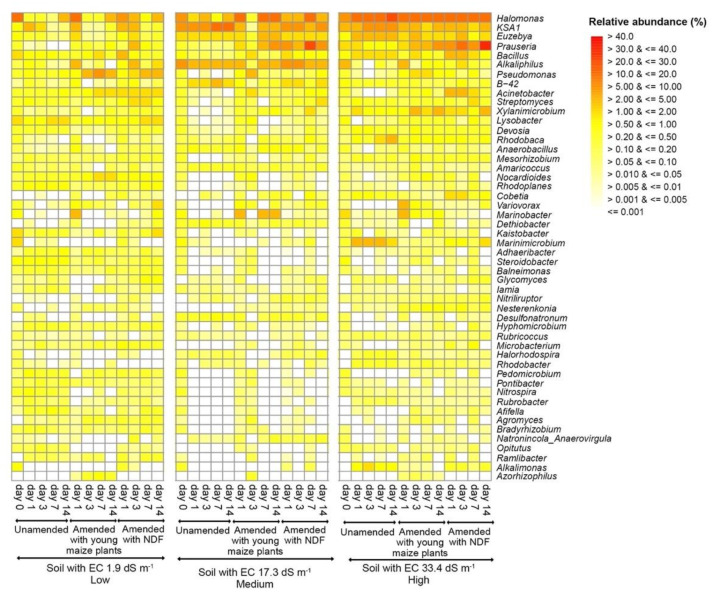
Heatmap with the relative abundance (%) of the 50 most abundant genera in the three soils from the former lake Texcoco with different electrolytic conductivity (EC) left unamended or amended with young maize plants and their neutral detergent fiber (NDF) fraction at the onset of the experiment (day 0) and incubated aerobically at 22 ± 2 °C for one (day 1), three (day 3), seven (day 7), or 14 days (day 14).

At the onset of the experiment, the unamended soil with EC 1.9 dS m^−1^ was dominated by the bacterial phyla Proteobacteria (55.44 ± 1.49% relative abundance), Actinobacteria (12.61 ± 1.87%), and the genera *Halomonas* (15.41 ± 13.22) and *Bacillus* (1.95 ± 0.73) (Figure 1 and Figure 2). In the unamended soil with EC 17.3 dS m^−1^, Proteobacteria (32.04 ± 22.04%), Firmicutes (15.21 ± 12.35%), *Halomonas* (9.27 ± 8.59%), and *Alkaliphilus* (9.24 ± 9.13%) were dominant phyla and genera. The unamended soil with EC 33.4 dS m^−1^ was dominated by Proteobacteria (40.95 ± 20.00%) and Actinobacteria (15.76 ± 8.12%), and the genera *Halomonas* (5.92 ± 1.81) and *KSA1* (4.29 ± 4.29%).

**Figure 3:**
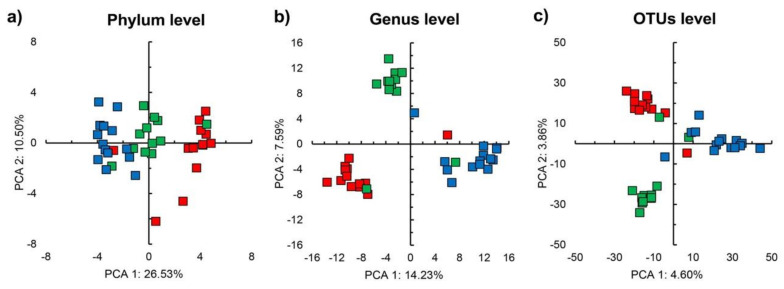
Principal component analysis (PCA) with centered log-ratio transformed count numbers of (**a**) all bacterial phyla, (**b**) all bacterial groups assigned up to the taxonomic level of genus and (**c**) all operational taxonomic units (OTUs) in unamended soil with electrolytic conductivity (EC) 1.9 dS m^−1^ (■) EC 17.3 dS m^−1^ (■), and EC 33.4 dS m^−1^ (■).

The PCA clearly separated the different unamended soils and clustered soil samples by EC (Figure 3a–c). The perMANOVA analysis confirmed that soil had a significant effect on the bacterial community structure, but not incubation time or the interaction between incubation time and soil (*p* < 0.05) (Table 2). The differential abundance analysis with aldex.kw showed that a large number of bacterial genera were significantly affected by soil, e.g., *Prauseria*, *Halomonas,* and *KSA1* (Table S3).

### 3.4. Bacterial Community Structure in the Organic Material Amended Soil

**Figure 4:**
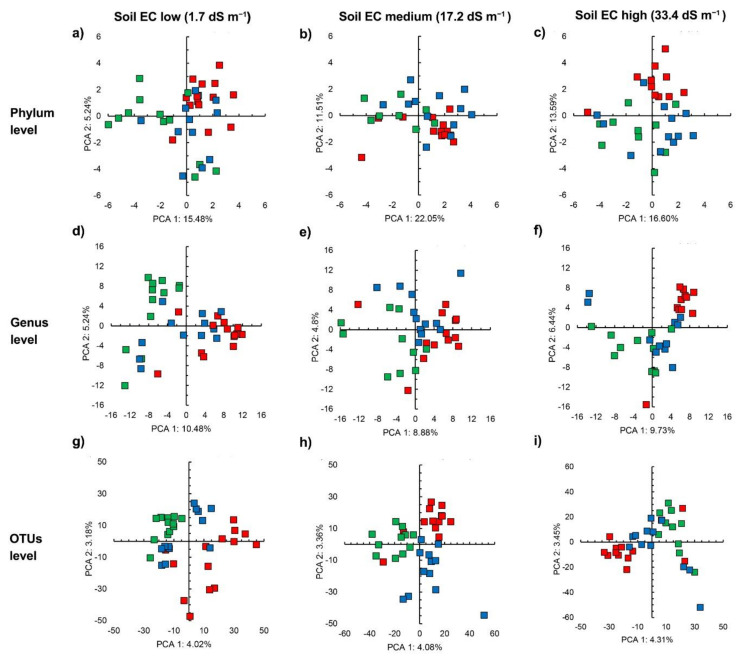
Principal component analysis (PCA) with the centered log-ratio transformed counts of bacterial groups in soil left unamended (■), or amended with NDF fraction (■) or young maize plants (■) at the phylum taxonomic level in soil with (**a**) electrolytic conductivity (EC) 1.9 dS m^−1^, (**b**) EC 17.3 dS m^−1^, and (**c**) EC 33.4 dS m^−1^, at the genus taxonomic level in soil with (**d**) EC 1.9 dS m^−1^, (**e**) EC 17.3 dS m^−1^, and (**f**) EC 33.4 dS m^−1^, and at the OTU level in soil with (**g**) EC 1.9 dS m^−1^, (**h**) EC 17.3 dS m^−1^, and (**i**) EC 33.4 dS m^−1^, incubated aerobically at 22 ± 2 °C for 14 days.

The application of young maize plants and their NDF fraction altered the soil bacterial community structure (Figure 4). The PCA clearly separated the maize that was amended from the unamended soil was the most accentuated when considering all OTUs (Figure 4g–i). The perMANOVA analysis showed that the application of organic material had a significant effect on the bacterial community structure, irrespective of soil or taxonomic level considered (Table 3). Time only affected the bacterial community structure when all bacterial groups assigned up to the taxonomic level of genus were considered. The differential abundance analysis with aldex.kw showed that a large number of bacterial genera were significantly affected by organic material application with the largest number in soil with highest EC and the least in soil with the lowest EC (Table 4).

**Figure 5:**
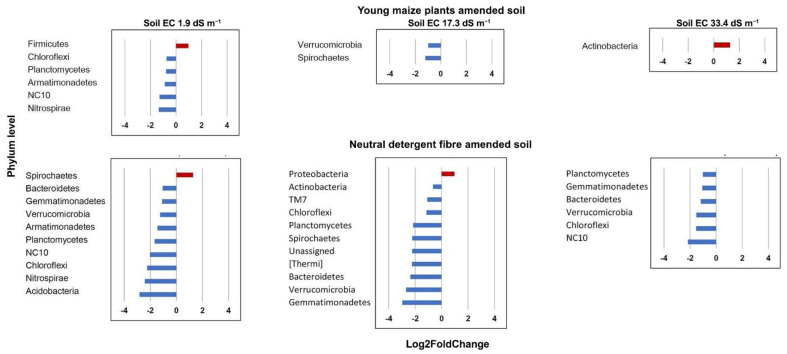
Differential abundance analysis with DESEq2 package [42]. A positive Log2FoldChange value (red bar) indicates a phylum that was significantly enriched by the application of young maize plants or its neutral detergent fibre (NDF) fraction, whereas a negative one (blue bar) indicates that the relative abundance of the phylum was significantly higher in unamended control soil. Only significantly affected bacterial groups at the taxonomic level of phylum are shown based on the Wald’s test (*p* < 0.05).

**Figure 6:**
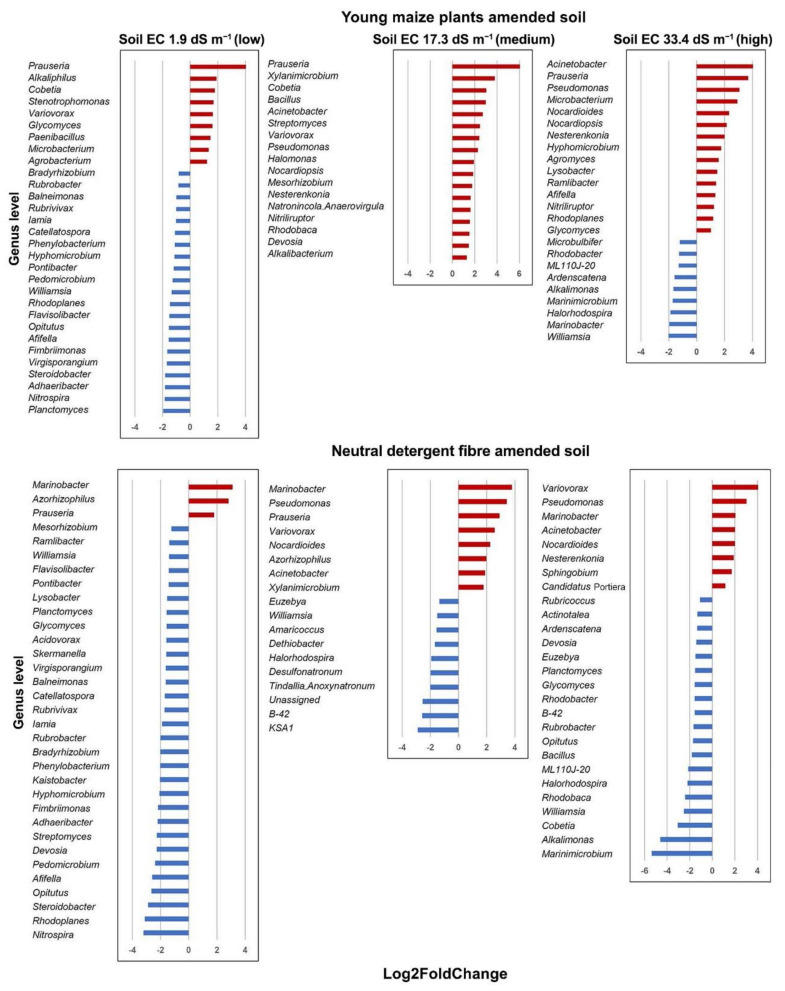
Differential abundance analysis with DESEq2 package [42]. A positive Log2FoldChange value (red bar) indicates a bacterial genus that was significantly enriched by the application of young maize plants or its NDF fraction, whereas a negative one (blue bar) indicates a bacterial genus that was significantly enriched in unamended control soil. Only significantly affected bacterial groups at the taxonomic level of genus are shown based on the Wald’s test (*p* < 0.05).

**Figure 7:**
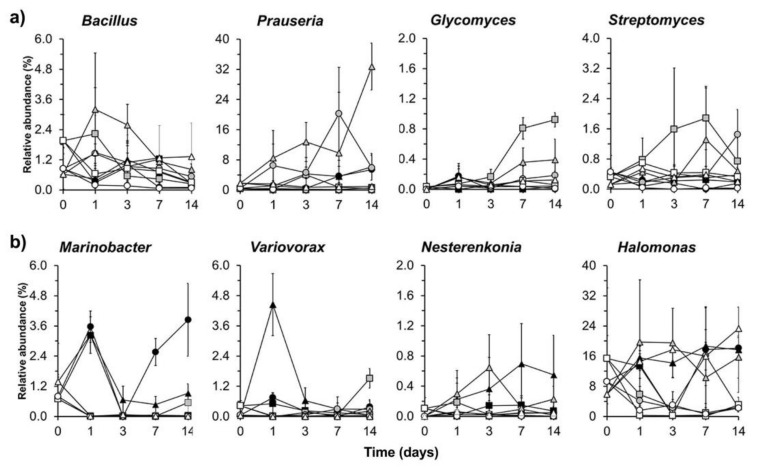
(**a**) Changes in the relative abundance (%) of bacterial genera with higher relative abundance in NDF amended soil than in the unamended soil incubated aerobically at 22 ± 2 °C for 14 days. Unamended soil with EC 1.9 dS m^−1^ (□), unamended soil with EC 17.3 dS m^−1^ (Δ), unamended soil with EC 33.4 dS m^−1^ (○), neutral detergent fibre fraction amended soil with EC 1.9 dS m^−1^ (■), neutral detergent fibre fraction amended soil with EC 17.3 dS m^−1^ (▲), neutral detergent fibre fraction amended soil with EC 33.4 dS m^−1^ (●), young maize plants amended soil with EC 1.9 dS m^−1^ (■), young maize plants amended soil with EC 33.4 dS m^−1^ (▲), young maize plants amended soil with EC 33.4 dS m^−1^ (●), and (**b**) changes in the relative abundance (%) of bacterial genera with higher relative abundance in the young maize plants amended soil than in the control soil that was incubated aerobically at 22 ± 2 °C for 14 days. Same legend as the subsection (**a**).

A sequence of bacterial genera was significantly enriched by the application of young maize plants and the NDF fraction when compared to the unamended soil (*p* < 0.05) (Figure 5 and Figure 6). Members of *Bacillus* were enriched in the maize amended soil with high EC at day 1 and *Prauseria* on day 3, while phylotypes to *Glycomyces* in soil with low and medium EC on day 7 and *Streptomyces* on day 14 (Figure 7a). In the NDF amended soil, members of *Marinobacter* were enriched on day 1, *Variovorax* on day 3, and *Nesternkonia* and *Halomonas* on day 7 (Figure 7b).

The PCA separated the soils with different EC when amended with young maize plants and its NDF fraction. The bacterial community in the NDF-amended soil with EC 1.9 dS m^−1^ was different from the one in soil with EC 33.4 dS m^−1^ when considering all of the bacterial groups assigned up to the taxonomic level of genus and when all the OTUs were considered (Figure S5a–c). Consequently, the perMANOVA analysis showed that soil had a significant effect on the bacterial community structure at the OTUs level when amended with NDF, but not incubation time (*p* < 0.05) (Table S4). The application of NDF to soil significantly affected members within *Xylanimicrobium* (*p* < 0.05) (Table S3).

The application of young maize plants clearly separated the different soils, as visualized by the PCA while considering all bacterial groups that were assigned up to the taxonomic level of genus and OTUs, but not considering all phyla (Figure S5d–f). As such, the perMANOVA analysis (Table S4) showed that soil had a strong significant effect on the bacterial community structure (*p* < 0.001). The analysis with ALDEx.kw showed that the relative abundance of a wide range of bacterial genera was significantly different between the soils that were amended with young maize plants, e.g., *KSA1*, *Bacillus*, *Alkaliphilus*, and *Halomonas* (Table S3).

The PCA considering all soils and treatments clearly separated the soils, while the effect of organic material on the bacterial community structure was smaller (Figure S6a,b). The aldex.kw analysis showed that soil had a highly significant effect on a larger number of bacterial groups than the application of young maize plants and its NDF fraction (*p* < 0.05) (Table S5).

### 3.5. Bacterial Functional Prediction Analysis

Proteobacteria showed the highest potential gene contribution to cellulose degradation (e.g., K01188, K01179), lignin degradation (e.g., K00428, K00432, K03863), hemicellulose degradation (e.g., K01181, K01198, K01206), and cello-oligosaccharides degradation (e.g., K01194, K11200) (Figure S7a). Actinobacteria and Firmicutes were the second most important gene contributors, being mainly related to cellulose, hemicellulose, and cello-oligosaccharides degradation. *Prauseria* and *Marinimicrobium* contained potential functions that were related to cellulose (K01225 and K01199) and hemicellulose degradation (K01218 and K01224) (Figure S7b). The largest number of potential genes was detected in soil with low EC and the least in soil with medium EC. The FAPROTAX analysis indicated that the number of potential metabolic functions related to the carbon and nitrogen cycles was larger in soil with low EC than in the other soils (Figure S8). Functions that were related to cellulose and lignin degradation were found in the three soils, i.e., cellulolysis, xylanolysis, and chitinolysis.

## 4. Discussion

Salinity affects the soil microbial communities due to osmotic pressure [43], low nutrient content [44], poor soil structure [45], and/or high pH [46]. The application of organic matter improves soil physical properties and increases the availability of nutrients [47]. Different types of organic material have been applied to saline soil to study how they might affect soil characteristics [48]. The application of organic material generally increased N, P, and K availability [49], while pH [50] and EC decreased [51]. Soil Bacteria play an important role in the degradation of organic material, so their involvement in the degradation of young maize plants and NDF fraction were studied in three soils from the former lake of Texcoco with a gradient of salinity and alkalinity. The NDF fraction of the maize plants was included in this study, as it might indicate which bacteria can degrade cellulose in alkaline saline soils.

### 4.1. Bacterial Community in Unamended Soil with Varying pH and EC

The bacterial community structure was clearly different in the three different soils of the former lake Texcoco. It can be assumed that soil characteristics, such as pH, particle size distribution, organic matter content, and EC, affect the relative abundance of the different bacterial groups. Although the soil bacterial community in Texcoco was different due to pH and EC, Proteobacteria and Actinobacteria both dominated this extreme environment. Keshri et al. [52] reported that Proteobacteria and Actinobacteria were also dominant in a soil with EC 23.7 dS m^−1^ and pH 9.1. Some members of the Proteobacteria are dominant in saline bare land, sewage-impacted, and lacustrine soil, as they thrive in high salinity and high pH environments [53,54]. In this study, *Halomonas* (Gammaproteobacteria) was the dominant genus in unamended alkaline saline soil. Phylotypes belonging to this genus have been found in a hypersaline lakebed [55], solonchak [56], and saline desert soils [57]. Previously, Oueriaghli et al. [58] studied the diversity and distribution of *Halomonas* in Rambla Salada, a hypersaline environment in Spain, and found higher diversity in soil with higher salinity.

Members of Actinobacteria, the second dominant phylum, have been reported to tolerate salinity, high and low temperatures, and low nutrient content [59]. For instance, *Prauseria* comprise various haloterant members [11,60]. In this study, the relative abundance of *Prauseria* increased from 0.07 ± 0.15% in soil with low EC (1.9 dS m^−1^) to 1.20 ± 0.47% in the soil with high EC (33.4 dS m^−1^). The members of *KSA1* (Bacteroidetes) were also enriched in soil with high salinity when compared to the soil with a low EC. Members of *KSA1* have been identified in a maar lakebed high pH and extreme salinity, which indicate a tolerance to these extreme conditions [61]. In contrast, members of Acidobacteria were negatively affected by soil salinity. The negative effect of salinity on members of Acidobacteria has been observed in a saline semi-arid soil [4].

It has to be remembered that not only were salinity and pH different between the soils, but soil organic matter content and particle size distribution also varied, which is known to affect the bacterial community. Soil with high sand content is characterised by its limited capacity to retain water and nutrients, while a soil with high clay content retains them [62]. Additionally, the bacteria are affected differently by the particle size distribution and soil water content [63]. Organic matter affects soil aggregation, aeration, and water movement, but also provides a C substrate and nutrients for microorganisms and plants [64]. Large amounts of easy decomposable organic material in soil favours copiotrophs, while nutrient limited environments enrich oligotrophs [65].

### 4.2. Bacterial Community Structure in Organic Material Amended Soil

The application of young maize plants or the NDF fraction profoundly changed the soil bacterial community structure. The NDF fraction, mainly (hemi)cellulose and lignin, is more resistant to degradation than the rest of the young maize plants, e.g., easy decomposable carbohydrates and proteins, and promotes lignocellulolytic bacterial groups [66]. In addition, the Texcoco soil is characterised by a low mineral N content [6]. The addition of NDF with a large amount of (hemi)cellulose and lignin and the limited availability of mineral N hindered the rapid assimilation of carbon, which, in turn, favoured some bacterial phylotypes, i.e., *Bacillus* (Firmicutes), *Prauseria* (Actinobacteria), *Glycomyces* (Actinobacteria), and *Streptomyces* (Actinobacteria).

Members of Actinobacteria, the second most abundant phylum, are involved in the degradation of lignin, cellulose, chitin and starch [67]. The functional prediction with PICRUSt showed that members of Actinobacteria, e.g., *Prauseria* and *Gordonia,* were the second most important contributors to cellulose and hemicellulose degradation. Little is known regarding the genus *Prauseria*. It was previously found to be enriched when young maize plants or its NDF fraction were applied to soil from the former lake Texcoco and might suggest participation in the degradation of lignocellulose [11]. Phylotypes that belong to *Gordonia* can degrade xenobiotics, environmental pollutants, and natural polymers [68]. Members of Bacilli, i.e., *Bacillus*, *Paenibacillus*, and *Anaerobacillus* were important contributors in the cellulose, hemicellulose, and cello-oligosaccharides degradation process [69,70,71]. Strains of *Bacillus* produce enzymes, such as cellulases, hemicellulases, and β-glucanases [69,72], whereas some members of *Paenibacillus* produce glucanases, chitinases, and cellulases that are involved in the degradation of organic material [70]. The enzymatic machinery of these bacterial groups suggests a metabolic specialization and an active participation in the degradation of high complexity C-compounds in the Texcoco soil. Members of *Anaerobacillus* were identified in hypersaline lakes [71], which indicated that they can thrive in saline environments, such as soil of the former lake Texcoco.

The addition of young maize plants to soil altered the bacterial community differently than when NDF was applied as the “soluble fraction”, i.e., the main component of young maize plants, was removed from the latter. The soluble fraction of plant residue comprises non-polymeric carbohydrates and proteins, and are more rapidly degraded by soil microorganisms [66]. The young maize plants that were applied to soil contained nearly 50% of easily decomposable organic material and had a higher N content than the NDF fraction. As such, young maize plants should enrich more members of bacteria with a copiotroph lifestyle than the NDF fraction, e.g., *Marinobacter* (Gammaproteobacteria), *Variovorax* (Betaproteobacteria), *Nesterenkonia* (Actinobacteria), and *Halomonas* (Gammaproteobacteria). Interestingly, these three bacterial groups also possess the ability to enhance plant growth [73,74,75].

Although the lignin content in the organic material applied to soil was low (<63 g kg^−1^ dry plant material), the functional prediction analysis showed that members of Alphaproteobacteria, Gammaproteobacteria, and Deltaproteobacteria were the primary gene contributors of the degradation of lignin, i.e., *Halomonas, Methylobacterium* and *Corallococcus*. Whilst *Marinimicrobium*, *Pseudomonas*, and *Phenylobacterium* were involved in the cellulose degradation in the Texcoco amended soils. Members of *Halomonas* can degrade lignocellulose (e.g., wheat straw) [76], and it is a source of lignocellulolytic haloenzymes [77]; it has also been found in alkaline-saline environments, including the Texcoco soil [78,79]. *Methylobacterium* is a methylotroph group participating in methane and methanol oxidation [80], as well as in the lignin degradation [81] and *Corallococcus* member has previously been found in saline-alkaline soils [82]. The members of *Marinimicrobium, Phenylobacterium,* and *Pseudomonas* are halotolerant and they possess the enzymatic machinery to degrade cellulose and lignin [83,84]. As such, members of Alphaproteobacteria, Gammaproteobacteria and Betaproteobacteria could be playing a major role in complex organic compounds degradation in alkaline-saline environments, such as the Texcoco soil.

Our results indicated that application of young maize plants or its NDF fraction enriched various groups. However, the extent of their enrichment might depend on other factors than just the application of organic material, e.g., soil edaphic variables. For instance, salinity changed the bacterial community structure in soil, and extreme salinity strongly reduced their diversity [85,86]. Bacterial groups with a known tolerance to soil salinity were enriched when young maize plants and the NDF fraction were applied, e.g., *Marinobacter* and *Prauseria*. Bacterial groups, such as *Mesorhizobium, Alkalibacterium*, *Microbacterium,* and *Hyphomicronium* were enriched when young maize plants were applied to soil, which indicates their participation in the degradation of lignocellulolytic material. These genera belong to the three phyla, i.e., Proteobacteria, Actinobacteria, and Firmicutes, often being dominant in saline soils [86].

Furthermore, it must be remembered that other microorganisms actively participate in the degradation of organic material in soil, e.g., fungi. Fungi are capable of metabolizing complex organic molecules, as reported extensively so they play an important role in the degradation of organic material in soil [87]. Further research might include a more comprehensive investigation of soil microbial community, i.e., including fungi, bacterial, protists, and viral community structure, using shotgun metagenomics. The assembly, as well as functional and taxonomical annotation, may increase our knowledge on how the soil microbial community in an extreme alkaline-saline soil responds to organic material input.

## 5. Conclusions

The soil bacterial community structure was profoundly altered by the application of both young maize plants and its NDF fraction. The application of easily decomposable organic material increased the relative abundance of members within Proteobacteria (mainly Gammaproteobacteria, e.g., *Acinetobacter* and *Pseudomonas*), Firmicutes (e.g., *Alkaliphilus* and *Paenibacillus*), and Actinobacteria (mainly *Prauseria*) when compared to the unamended soil. Young maize plants induced larger changes in soil bacterial community than the application of the NDF fraction, as the young maize plants contained easily decomposable organic material in the “soluble fraction”, which was absent in the NDF fractions. The NDF fraction mostly contained (hemi)cellulose and lignin, which are more difficult to mineralize. The soil bacterial functional prediction confirmed that, irrespective of the high salinity and pH in soil of the former lake Texcoco, the application of organic material increased the relative abundance of bacterial groups with the capacity to mineralize high and complex C compounds, e.g., lignin and (hemi)cellulose.

## Figures and Tables

**Table 1:** Characteristics of the three locations used in this study.

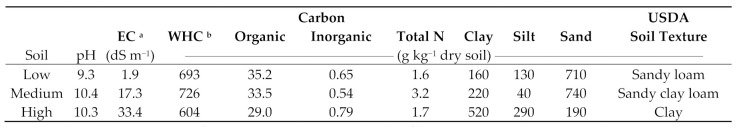

^a^ EC: Electrolytic conductivity, ^b^ WHC: Water holding capacity.

**Table 2:** The effect of soil (EC 1.9 dS m^−1^, EC 17.3 dS m^−1^ and EC 33.4 dS m^−1^), time (0, 1, 3, 7 and 14 days of aerobic incubation) and their interaction on the soil bacterial community using a perMANOVA analysis with the sequence counts converted to centered log-ratio transformation with ALDEx2 package [39].

Comparison	F Value	*p* Value ^a^
Phylum		
Soil	4.071	**<0.001**
Time	1.503	0.080
Interaction	1.143	0.273
All taxonomic groups assigned to the taxonomic level of genus		
Soil	3.865	**<0.001**
Time	0.996	0.396
Interaction	1.059	0.284
All operational taxonomic units (OTUs)		
Soil	1.577	**<0.001**
Time	1.003	0.364
Interaction	0.992	0.579

^a^*p*-values were determined using 999 permutations. Bold *p*-values indicate a significant effect.

**Table 3:** The effect of treatment (unamended, young maize plants amended soil or neutral detergent fibre amended soil), time (one, three, seven, and 14 days of aerobic incubation) and their interaction on the soil bacterial community, as determined with a perMANOVA analysis with the sequence counts converted to centeredlog-ratio transformation with ALDEx2 package [39].

	Soil EC 1.9 dS m^−1^		Soil EC 17.2 dS m^−1^		Soil EC 33.4 dS m^−1^	
Comparison	F Value	*p* Value ^a^	F Value	*p* Value	F Value	*p* Value
Phyla						
Treatment	2.070	**<0.001**	1.551	**<0.019**	1.848	**<0.001**
Time	1.003	0.434	1.261	0.197	1.242	0.174
Interaction	1.028	0.412	1.446	0.065	1.062	0.341
All Taxonomic Groups Assigned up to the Level of Genus						
Treatment	1.829	**<0.001**	1.560	**<0.001**	1.556	**<0.001**
Time	1.148	**<0.018**	1.185	**<0.004**	1.191	**<0.004**
Interaction	1.101	**<0.031**	1.135	**<0.004**	0.968	0.723
All Operational Taxonomic Units (OTUs)						
Treatment	1.179	**<0.001**	1.184	**<0.001**	1.196	**<0.001**
Time	1.021	0.171	1.025	0.146	0.989	0.621
Interaction	0.995	0.568	1.023	0.102	1.015	0.219

^a^*p*-values are based on 999 permutations. Bold *p*-values indicate a significant effect.

**Table 4:** The effect of treatment (unamended soil or soil amended with NDF or soil amended with young maize plants) on the soil bacterial community structure at the phyla and genus taxonomic level in soil with EC 1.9 dS m^−1^ (low), EC 17.3 dS m^−1^ (medium), and EC 33.4 dS m^−1^ (high) using a compositional approach, i.e., the analysis of differential abundance taking sample variation into account with the ALDEx2 package [39].

Soil EC 1.9 dS m^−1^		Soil EC 17.3 dS m^−1^		Soil EC 33.4 dS m^−1^	
Phyla					
Taxonomic group	*p* Value ^a^	Taxonomic group	*p* Value	Taxonomic group	*p* Value
Acidobacteria	0.0076	Verrucomicrobia	0.0062	Actinobacteria	0.0365
Chloroflexi	0.0105	Gemmatimonadetes	0.0039		
Nitrospirae	0.0140	Proteobacteria	0.0073		
Proteobacteria	0.0199	Bacteroidetes	0.0089		
NC10	0.0424	Planctomycetes	0.0190		
Genera					
*Pseudomonas*	0.0025	*Prauseria*	0.0006	*Marinobacter*	0.0133
*Azorhizophilus*	0.0026	*KSA1*	0.0059	*Acinetobacter*	0.0154
*Rhodoplanes*	0.0037	*Streptomyces*	0.0200	*Marinimicrobium*	0.0195
*Nitrospira*	0.0063	*Bacillus*	0.0279	*Bacillus*	0.0303
*Opitutus*	0.0099	*B-42*	0.0299	*Alkalimonas*	0.0317
*Steroidobacter*	0.0102			*Cobetia*	0.0351
*Acinetobacter*	0.0125			*Variovorax*	0.0405
*Glycomyces*	0.0132				
*Streptomyces*	0.0136				
*Prauseria*	0.0196				
*Devosia*	0.0367				
*Nocardioides*	0.0400				

^a^ Significance was measured using ALDEx2 argument within ALDEx2 package [39] and using the Benjamini-Hochberg correction procedure of the Kruskal-Wallis test (significance threshold, *p* < 0.05).

## Data Availability

Sequence data have been deposited in the NCBI Sequence Read Archive (SRA) under accession refence PRJNA722365.

## Supplementary Materials

The following are available online at https://www.mdpi.com/article/10.3390/microorganisms9061297/s1, Figure S1: Schematic overview of the soil sampling procedure, Figure S2: Cumulative emission of CO_2_ (mg C kg^−1^ dry soil) from unamended soil with EC 1.9 dS m^−1^ (□), unamended soil with EC 17.3 dS m^−1^ (Δ), unamended soil with EC 33.4 dS m^−1^ (○), neutral detergent fibre fraction amended soil with EC 1.9 dS m^−1^ (■), neutral detergent fibre fraction amended soil with EC 17.3 dS m^−1^ (▲), neutral detergent fibre fraction amended soil with EC 33.4 dS m^−1^ (●), young maize plants amended soil with EC 1.9 dS m^−1^ (■), young maize plants amended soil with EC 33.4 dS m^−1^ (▲), young maize plants amended soil with EC 33.4 dS m^−1^ (●) incubated aerobically at 22 ± 2 °C for 14 days, Figure S3: Rarefaction curve or the relation between the number of sequences analysed and operational taxonomic units (OTUs) obtained. Unamended soil with EC 1.9 dS m^−1^ (□), unamended soil with EC 17.3 dS m^−1^ (Δ), unamended soil with EC 33.4 dS m^−1^ (○), neutral detergent fibre fraction amended soil with EC 1.9 dS m^−1^ (■), neutral detergent fibre fraction amended soil with EC 17.3 dS m^−1^ (▲), neutral detergent fibre fraction amended soil with EC 33.4 dS m^−1^ (●), young maize plants amended soil with EC 1.9 dS m^−1^ (■), young maize plants amended soil with EC 33.4 dS m^−1^ (▲), young maize plants amended soil with EC 33.4 dS m^−1^ (●), Figure S4: (a) Boxplot of the alfa diversity of the bacterial communities based on the equivalent Hill numbers at different *q* orders (at *q* = 0, 1 and 2) in the unamended soil with electrolytic conductivity (EC) 1.9 dS m^−1^ (low), EC 17.3 dS m^−1^ (medium) and EC 33.4 dS m^−1^ (high) at the onset of the experiment, and (b) changes in alfa diversity in the unamended soil with EC 1.9 dS m^−1^ (□), unamended soil with EC 17.3 dS m^−1^ (Δ), unamended soil with EC 33.4 dS m^−1^ (○), neutral detergent fibre fraction amended soil with EC 1.9 dS m^−1^ (■), neutral detergent fibre fraction amended soil with EC 17.3 dS m^−1^ (▲), neutral detergent fibre fraction amended soil with EC 33.4 dS m^−1^ (●), young maize plants amended soil with EC 1.9 dS m^−1^ (■), young maize plants amended soil with EC 33.4 dS m^−1^ (▲), young maize plants amended soil with EC 33.4 dS m^−1^ (●) incubated aerobically at 22 ± 2 °C for 14 days, Figure S5: Principal component analysis (PCA) with the centered log-ratio transformed counts of bacterial groups in soil with electrolytic conductivity (EC) 1.9 dS m^−1^ (■), EC 17.3 dS m^−1^ (■) and EC 33.4 dS m^−1^ (■) amended with the neutral detergent fibre (NDF) fraction at the taxonomic level of (a) all bacterial phyla, (b) all bacterial genera and (c) all bacterial OTUs, and PCA with the centered log-ratio transformed counts of bacterial groups in soil with electrolytic conductivity (EC) 1.9 dS m^−1^ (■), EC 17.3 dS m^−1^ (■) and EC 33.4 dS m^−1^ (■) amended with young maize plants at the taxonomic level of (d) all bacterial phyla, (e) all bacterial genera, and (f) all bacterial OTUs, incubated aerobically at 22 ± 2 °C for 14 days, Figure S6: (a) Principal component analysis (PCA) with clr-transformed count numbers of all phyla, all bacterial groups assigned up to the taxonomic level of genus and all operational taxonomic units (OTUs) in unamended soil with EC 1.9 dS m^−1^ (■), unamended soil with EC 17.3 dS m^−1^ (■), unamended soil with EC 33.4 dS m^−1^ (■), neutral detergent fibre fraction amended soil with EC 1.9 dS m^−1^ (●), neutral detergent fibre fraction amended soil with EC 17.3 dS m^−1^ (●), neutral detergent fibre fraction amended soil with EC 33.4 dS m^−1^ (●), young maize plants amended soil with EC 1.9 dS m^−1^ (▲), young maize plants amended soil with EC 17.3 dS m^−1^ (▲), young maize plants amended soil with EC 33.4 dS m^−1^ (▲) incubated aerobically at 22 ± 2 °C for 14 days and (b) the effect of treatment (left unamended, application of young maize plants or their NDF fraction), time and their interaction on the bacterial community structure with the permutational multivariate analyses of variance (perMANOVA) test, Figure S7: Functional prediction analysis with PICRUSt of genes involved in the cellulose, hemicellulose, lignin and cello-oligosaccharides degradation of bacterial groups assigned up to the taxonomic level of (a) phylum and (b) genus in soil of the former Lake of Texcoco aerobically incubated for 14 days. UN: unamended soil, YMR: young maize plants amended soil, NDF: neutral detergent fibre amended soil. Low: soil EC 1.9 dS m^−1^, medium: soil EC 17.3 dS m^−1^ and high: soil EC 33.4 dS m^−1^. Bacterial groups with gene contribution (relative abundance) > 0.5% are shown, Figure S8: Functional annotation of prokaryotic taxa (FAPROTAX) analysis showing functions that are related to (a) carbon and (b) nitrogen cycle in soil of the former lake of Texcoco. Shapes contrast different treatment: unamended soil (○), soil amended with neutral detergent fibre (NDF) (■) and soil amended with young maize plants (▲), Table S1. Characteristics of organic material used in the aerobic incubation experiment, Table S2. List of KOs used for the analysis of genes involved in cellulose, lignin, hemicellulose, and cello-oligosaccharides degradation, Table S3. Effect of soil (EC: 1.9 dS^−1^ (low), 17.3 dS^−1^ (medium), 33.4 dS^−1^ (high)) on bacterial phyla and genera in the unamended soil, soil amended with NDF and soil amended with young maize plants using a compositional approach, i.e., analysis of differential abundance taking sample variation into account ALDEx2 package [39], Table S4. Effect of soil (EC 1.9 dS m^−1^, EC 17.3 dS m^−1^, 33.4 dS m^−1^), time (one, three, seven, and 14 days of aerobic incubation) and their interaction on the soil bacterial community in the young maize plants and neutral detergent fibre (NDF) amended soil using a perMANOVA analysis with the sequence counts converted to centeredlog-ratio transformation with ALDEx2 package [39], Table S5. Effect of treatment (unamended soil, amended soil with young maize plants or its NDF) and effect of soil (EC: 1.9 dS m^−1^, 17.3 dS m^−1^, 33.4 dS m^−1^) on bacterial phyla or genera using a compositional approach, i.e., analysis of differential abundance taking sample variation into account (ALDEx2 package [39]).
