# Supplementary material for: Bacterial Communities in Alkaline Saline Soils Amended with Young Maize Plants or Its (Hemi)Cellulose Fraction
Source: Microorganisms. 2021 Jun 15;9(6):1297. doi: 10.3390/microorganisms9061297 (PMC8232260; doi:10.3390/microorganisms9061297)
Supplement: Supplementary file 1 [file microorganisms-09-01297-s001.zip › Table S1.pdf]

Table S1. Characteristics of organic material used in the aerobic incubation experiment [a].

	Soluble fraction	Lignin content	(Hemi) cellulose	Polyphenols	Ash	Total N	Total C
	(g kg^{-1} dry plant material)						
Young maize plants	599 (6.5) [b]	25 (1.1)	311 (8.0)	2 (0.2)	62 (2.3)	34 (0.6)	422 (1.9)
NDF [c]	0	63 (2.8)	776 (15.4)	6 (0.4)	155 (2.9)	19 (0.5)	405 (1.8)

[a] Data derived from Ramirez-Villanueva et al. [12], [b] Value between parentheses is the standard deviation; [c] NDF: Neutral detergent fibre.
