# Supplementary material for: Bacterial Communities in Alkaline Saline Soils Amended with Young Maize Plants or Its (Hemi)Cellulose Fraction
Source: Microorganisms. 2021 Jun 15;9(6):1297. doi: 10.3390/microorganisms9061297 (PMC8232260; doi:10.3390/microorganisms9061297)
Supplement: Supplementary file 1 [file microorganisms-09-01297-s001.zip › Table S3.pdf]

Table S3. Effect of soil with EC 1.9 dS $^{-1}$ (low), 17.3 dS $^{-1}$ (medium), 33.4 dS $^{-1}$ (high) on bacterial phyla and genera in the unamended soil, soil amended with neutral detergent fibre (NDF) and soil amended with young maize plants using a compositional approach, i.e. analysis of differential abundance taking sample variation into account ALDEx2 package [39].

**Unamended** [a]		**NDF treatment**		**Young maize plants**	
Prauseria	0.00009 [b]	*Xylanimicrobium*	0.0360	*KSA1*	0.0070
Rhodoplanes	0.0001			*Prauseria*	0.0084
KSA1	0.0001			*Lysobacter*	0.0089
Lysobacter	0.0001			*Alkaliphilus*	0.0094
Pseudomonas	0.0001			*Euzebya*	0.0121
Steroidobacter	0.0001			*Nocardioides*	0.0121
Halomonas	0.0001			*Halomonas*	0.0137
Euzebya	0.0001			*Devosia*	0.0156
B-42	0.0002			*B-42*	0.0160
Acinetobacter	0.0002			*Anaerobacillus*	0.0171
Rhodobaca	0.0002			*Pseudomonas*	0.0198
Devosia	0.0003			*Desulfonatronum*	0.0230
Desulfonatronum	0.0003			*Steroidobacter*	0.0240
Nocardioides	0.0003			*Dethiobacter*	0.0322
Cobetia	0.0003			*Bacillus*	0.0359
Pedomicrobium	0.0003			*Bradyrhizobium*	0.0360
Marinimicrobium	0.0004				
Alkaliphilus	0.0005				
Balneimonas	0.0005				
Adhaeribacter	0.0005				

[a] Only the twenty bacterial genera in the unamended soil most highly significantly affected $p < 0.001$. [b] Significance was measured using ALDEx2 and based on the Benjamini-Hochberg corrected p value of the Kruskal-Wallis test (significance threshold, $p < 0.05$).
