# Supplementary material for: Bacterial Communities in Alkaline Saline Soils Amended with Young Maize Plants or Its (Hemi)Cellulose Fraction
Source: Microorganisms. 2021 Jun 15;9(6):1297. doi: 10.3390/microorganisms9061297 (PMC8232260; doi:10.3390/microorganisms9061297)
Supplement: Supplementary file 1 [file microorganisms-09-01297-s001.zip › Table S4.pdf]

Table S4. Effect of soil (EC 1.9 dS m^{-1}, EC 17.3 dS m^{-1}, 33.4 dS m^{-1}), time (1, 3, 7 and 14 days of aerobic incubation) and their interaction on the soil bacterial community in the young maize plants and neutral detergent fibre (NDF) amended soil using a perMANOVA analysis with the sequence counts converted to centered-log ratio transformation with ALDEx2 package [39].

	NDF treatment [1]		Young maize plants	
Factor	F value	*p* Value	F value	*p* Value
		Phyla		
Soil	1.309	0.175	2.432	**< 0.001**
Time	0.989	0.496	1.470	0.086
Interaction	0.888	0.661	1.014	0.413
All taxonomic groups assigned to the level of bacterial genus				
Soil	1.103	0.139	1.987	**< 0.001**
Time	1.113	0.055	1.074	0.222
Interaction	1.051	0.227	0.963	0.610
All operational taxonomic units (OTUs)				
Soil	1.041	**< 0.012**	1.176	**< 0.001**
Time	1.014	0.230	1.056	0.058
Interaction	1.007	0.300	1.012	0.228

[1] NDF: natural detergent fibre, [2] *p*-values are based on 999 permutations, and bold values indicate a significant effect.
