# Supplementary material for: Bacterial Communities in Alkaline Saline Soils Amended with Young Maize Plants or Its (Hemi)Cellulose Fraction
Source: Microorganisms. 2021 Jun 15;9(6):1297. doi: 10.3390/microorganisms9061297 (PMC8232260; doi:10.3390/microorganisms9061297)
Supplement: Supplementary file 1 [file microorganisms-09-01297-s001.zip › Table S5.pdf]

Table S5. Effect of treatment (unamended soil, amended soil with young maize plants or its neutral detergent fibre) and effect of soil (EC: 1.9 dS m^{-1}, 17.3 dS m^{-1}, 33.4 dS m^{-1}) on bacterial phyla or genera using a compositional approach, i.e. analysis of differential abundance taking sample variation into account (ALDEx2 package [39]).

**Soil** [a]		**Treatment**	
Phyla			
Acidobacteria	2.99×10^{-10} [b]	Verrucomicrobia	1.00×10^{-4}
Firmicutes	9.63×10^{-8}	Proteobacteria	1.00×10^{-4}
Gemmatimonadetes	1.10×10^{-6}	Planctomycetes	3.00×10^{-4}
Bacteroidetes	5.77×10^{-7}	Actinobacteria	5.00×10^{-4}
[Thermi]	2.92×10^{-7}	Chloroflexi	8.00×10^{-4}
Proteobacteria	5.00×10^{-4}	Gemmatimonadetes	3.10×10^{-3}
Actinobacteria	3.00×10^{-4}	Bacteroidetes	4.80×10^{-3}
Armatimonadetes	1.00×10^{-4}	Nitrospirae	9.20×10^{-3}
Spirochaetes	1.70×10^{-3}	NC10	3.62×10^{-2}
Chloroflexi	1.30×10^{-3}		
Genera			
Halomonas	9.35×10^{-9}	*Acinetobacter*	3.14×10^{-7}
Lysobacter	1.10×10^{-8}	*Prauseria*	4.62×10^{-7}
KSA1	1.14×10^{-8}	*Variovorax*	4.32×10^{-6}
Euzebya	1.36×10^{-8}	*Streptomyces*	5.70×10^{-6}
Xylanimicrobium	2.10×10^{-8}	*Glycomyces*	6.60×10^{-6}
Alkaliphilus	3.37×10^{-8}	*Pseudomonas*	2.82×10^{-5}
Nocardioides	6.98×10^{-8}	*Marinobacter*	8.93×10^{-5}
Prauseria	1.15×10^{-7}	*Azorhizophilus*	2.00×10^{-3}
Pseudomonas	6.74×10^{-7}	*Williamsia*	1.00×10^{-3}
Anaerobacillus	6.77×10^{-7}	*Bacillus*	7.00×10^{-3}
Rhodobaca	9.60×10^{-7}	*Nitriliruptor*	4.70×10^{-3}
Steroidobacter	1.12×10^{-6}	*Nesterenkonia*	6.40×10^{-3}
Desulfonatronum	1.30×10^{-6}		
Devosia	1.53×10^{-6}		
B-42	2.67×10^{-6}		
Rhodoplanes	5.57×10^{-6}		
Dethiobacter	1.40×10^{-5}		
Hyphomicrobium	2.61×10^{-5}		
Pedomicrobium	4.11×10^{-5}		
Adhaeribacter	4.63×10^{-5}		

[a] Only ten phyla and twenty genera most significantly affected by soil EC $P < 0.001$ and with the lowest p values obtained are given, [b] The expected values of the Kruskal-Wallis test for each feature obtained with aldex.kw argument with converted sequence data using the centered log-ratio transform test returned by the aldex.clr argument ALDEx2 package.
