# Supplementary figures and images for: Bacterial Communities in Alkaline Saline Soils Amended with Young Maize Plants or Its (Hemi)Cellulose Fraction
Source: Microorganisms. 2021 Jun 15;9(6):1297. doi: 10.3390/microorganisms9061297 (PMC8232260; doi:10.3390/microorganisms9061297)

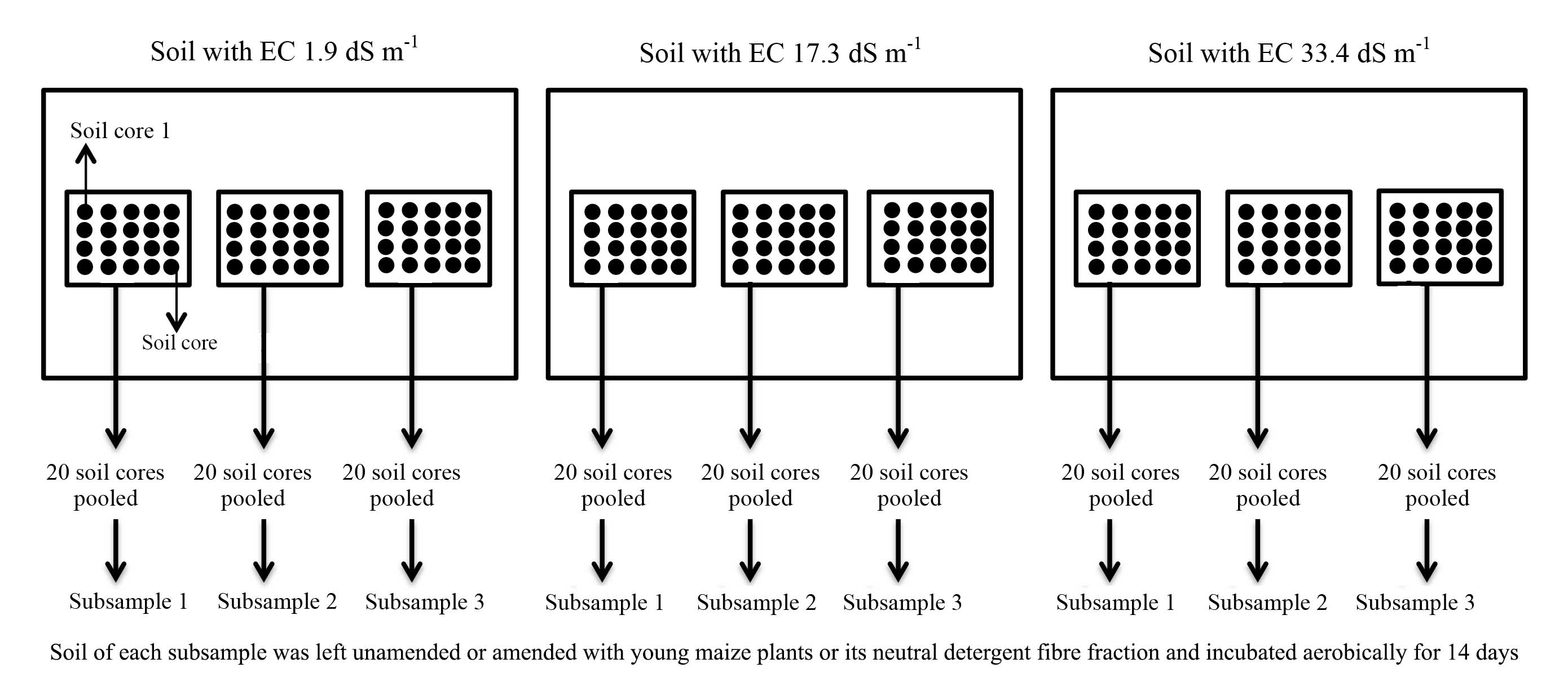

Supplement: Supplementary file 1 [file microorganisms-09-01297-s001.zip › Figure S1.jpg]

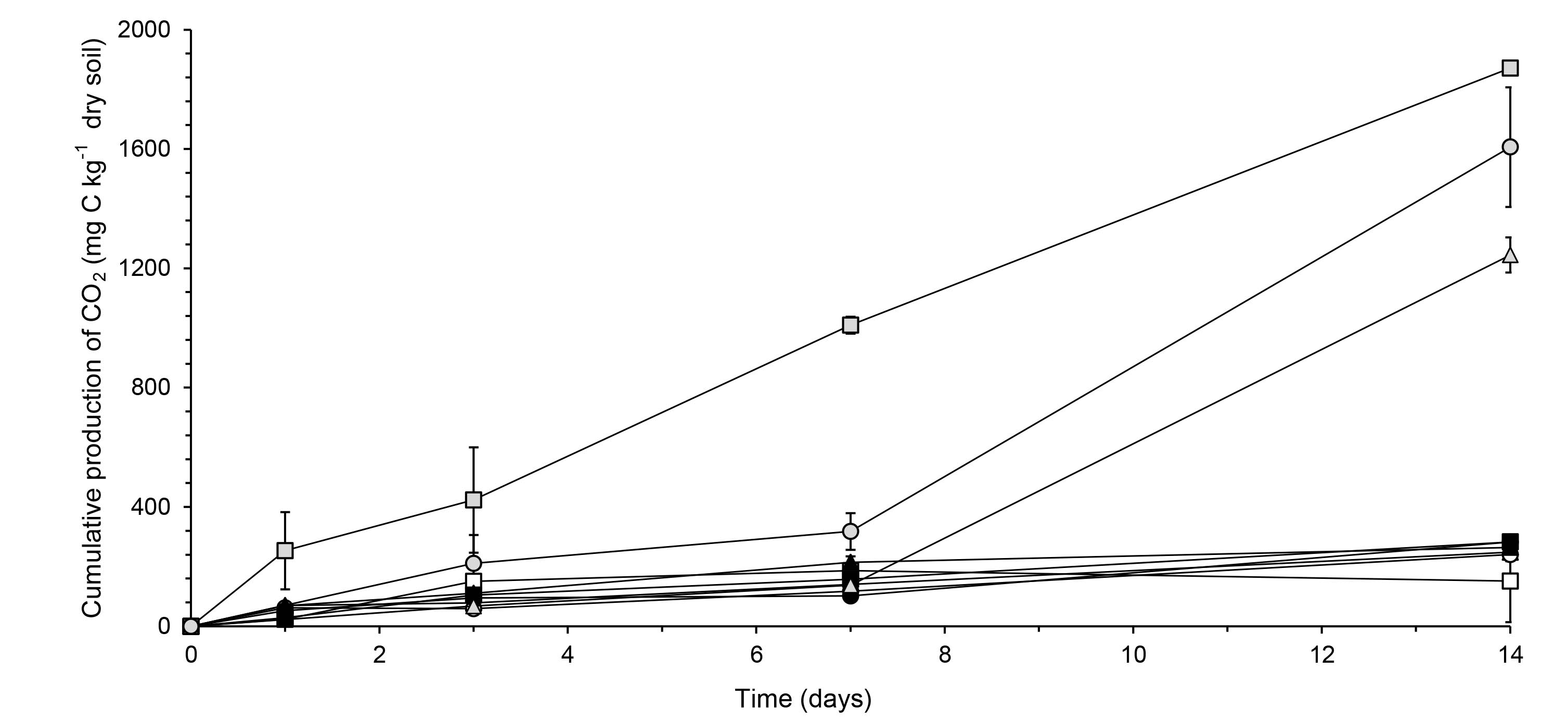

Supplement: Supplementary file 1 [file microorganisms-09-01297-s001.zip › Figure S2.jpg]

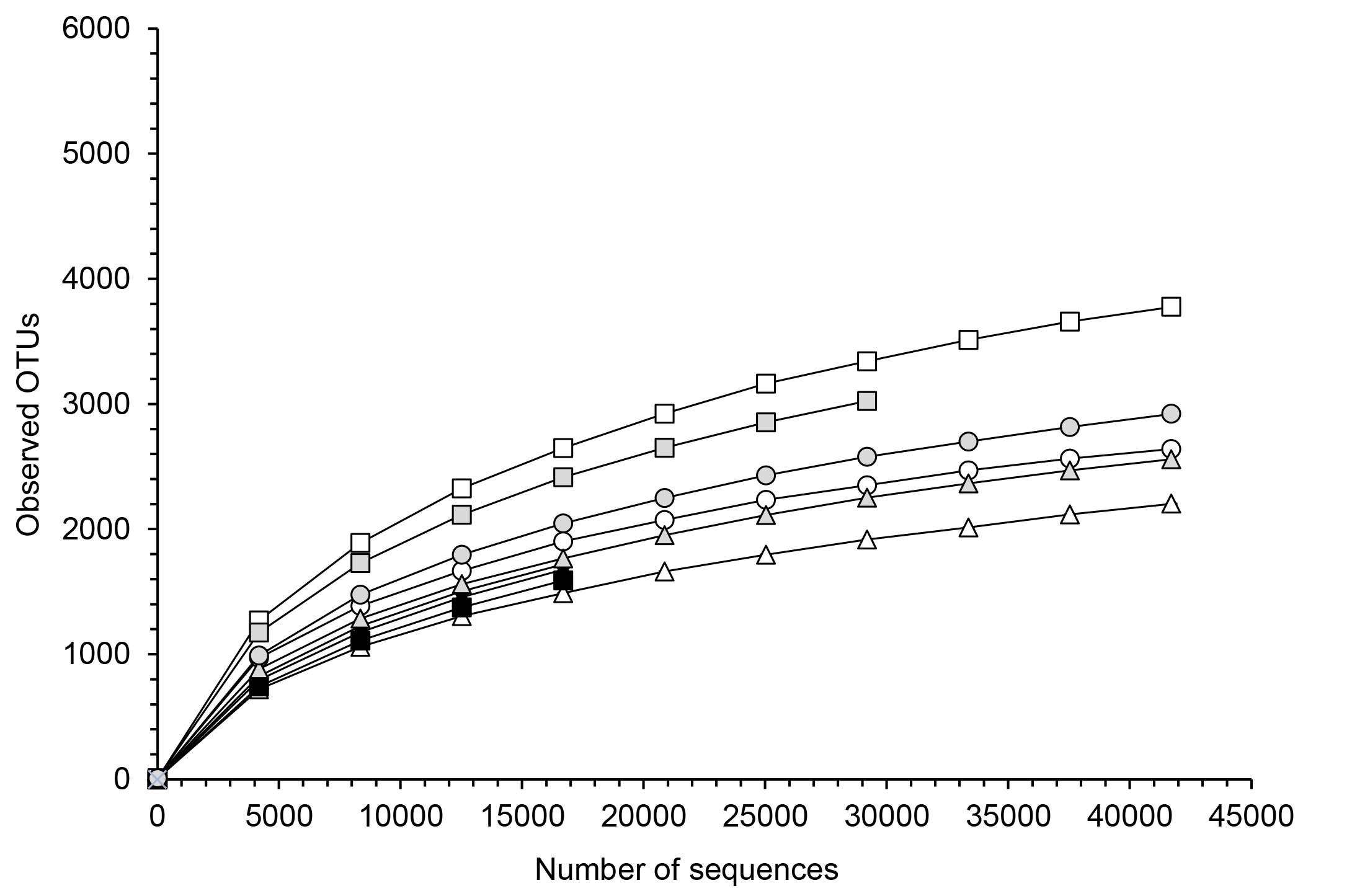

Supplement: Supplementary file 1 [file microorganisms-09-01297-s001.zip › Figure S3.jpg]

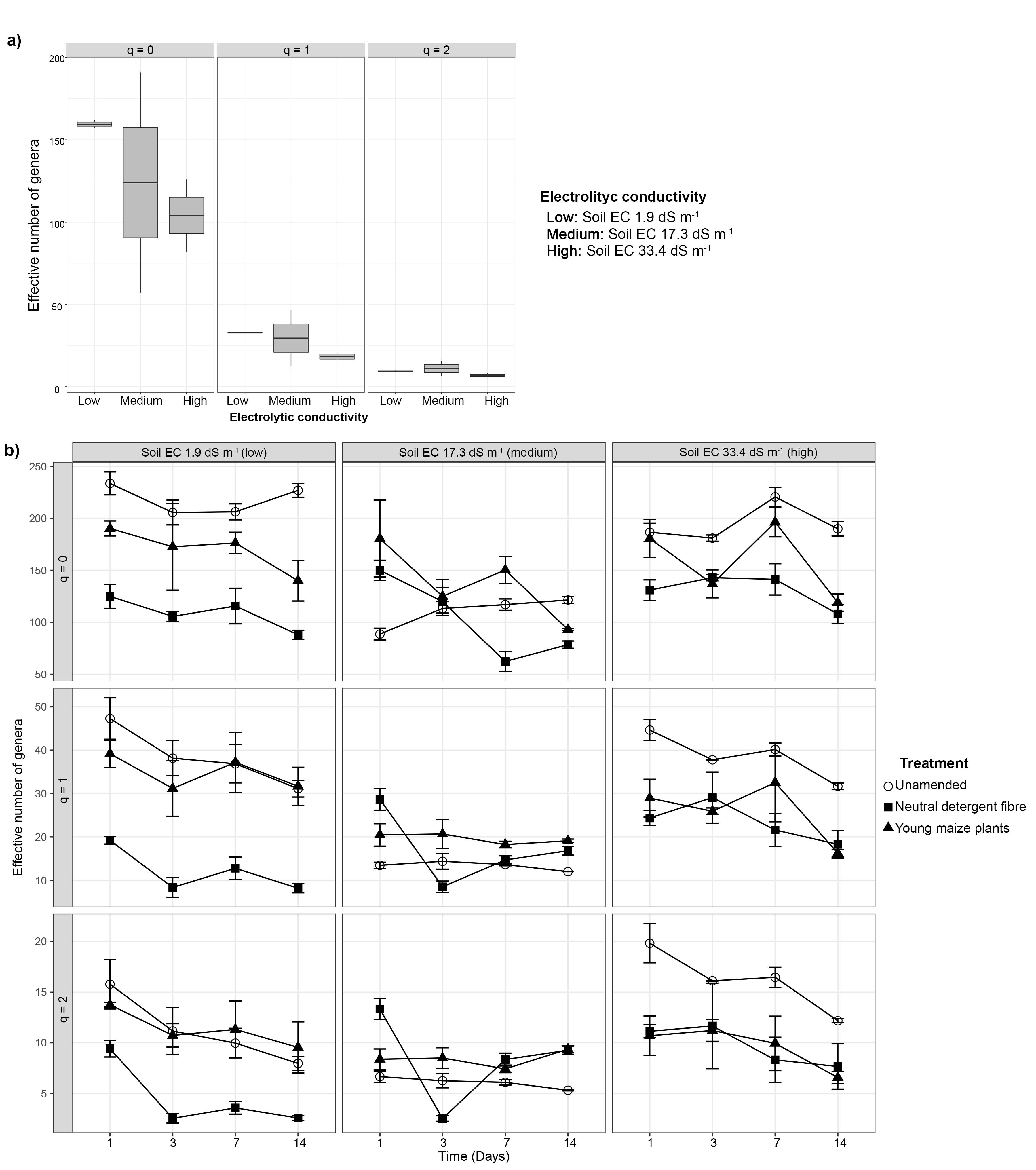

Supplement: Supplementary file 1 [file microorganisms-09-01297-s001.zip › Figure S4.jpg]

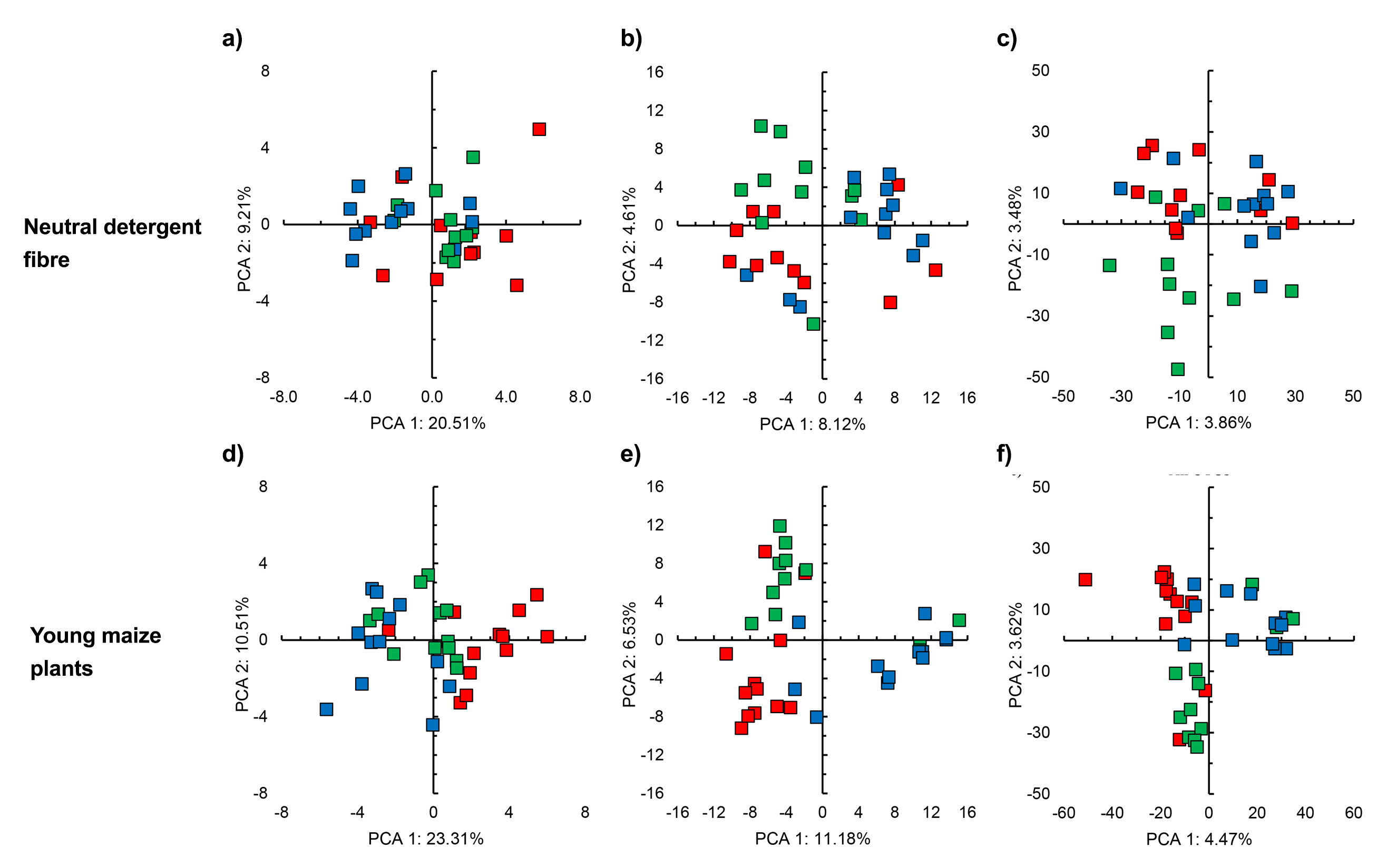

Supplement: Supplementary file 1 [file microorganisms-09-01297-s001.zip › Figure S5.jpg]

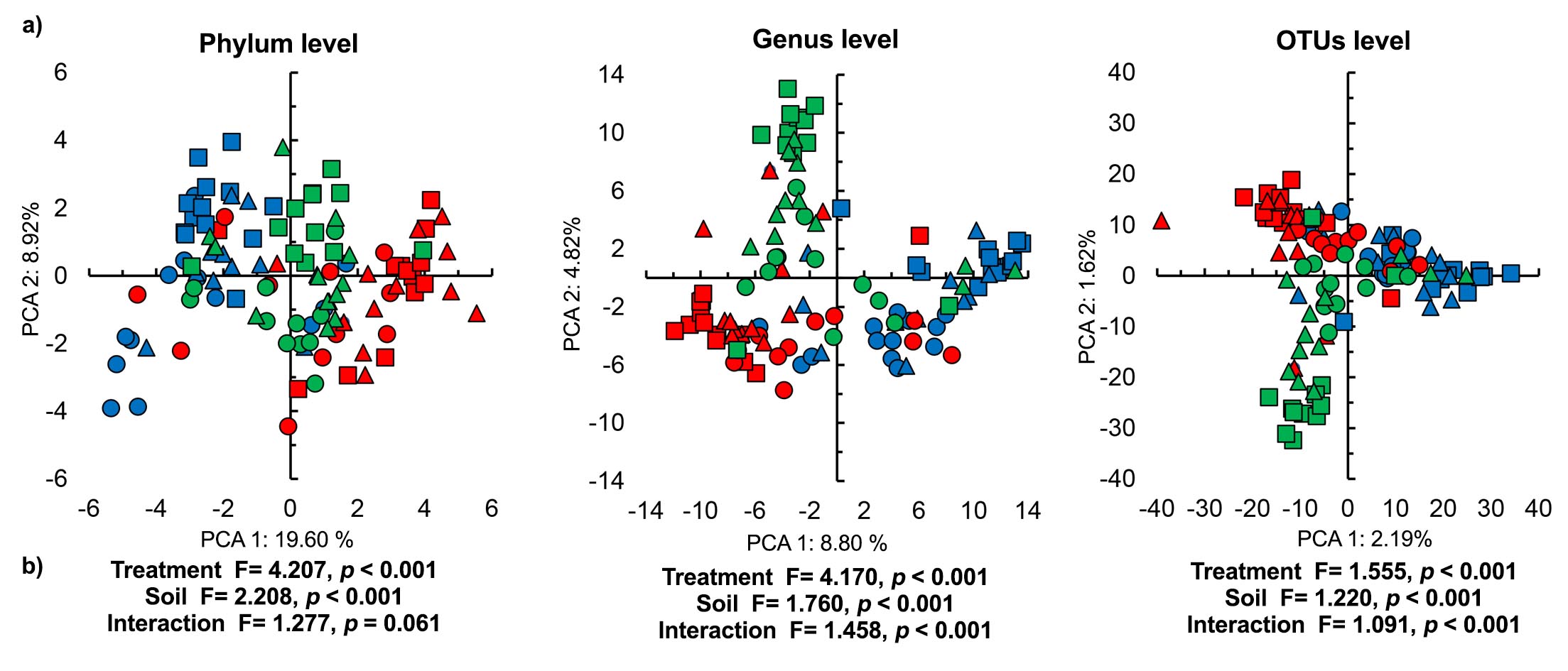

Supplement: Supplementary file 1 [file microorganisms-09-01297-s001.zip › Figure S6.jpg]

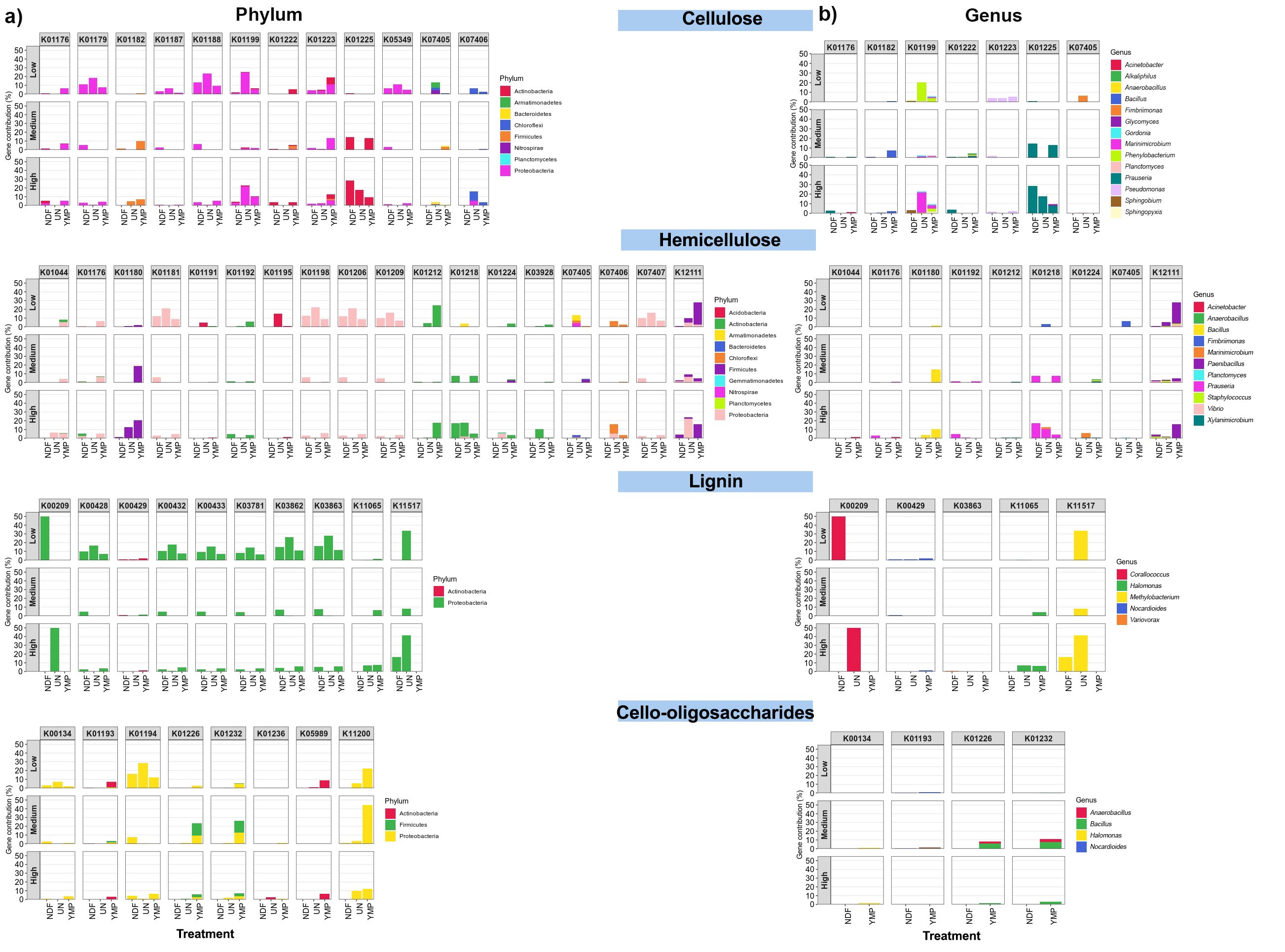

Supplement: Supplementary file 1 [file microorganisms-09-01297-s001.zip › Figure S7.jpg]

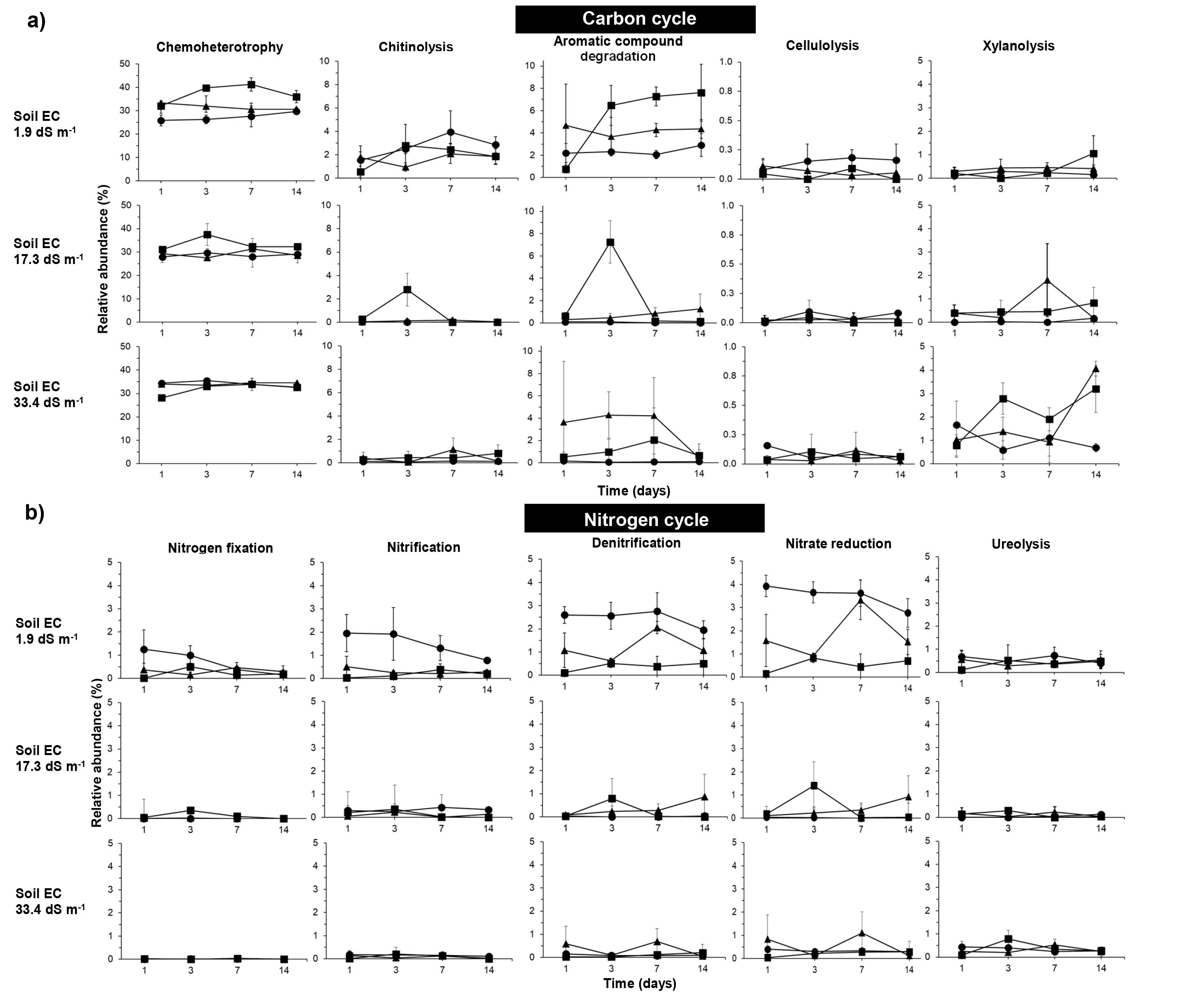

Supplement: Supplementary file 1 [file microorganisms-09-01297-s001.zip › Figure S8.jpg]
